# Uptake and fecal excretion of *Coxiella burnetii* by *Ixodes ricinus* and *Dermacentor marginatus* ticks

**DOI:** 10.1186/s13071-020-3956-z

**Published:** 2020-02-14

**Authors:** Sophia Körner, Gustavo R. Makert, Katja Mertens-Scholz, Klaus Henning, Martin Pfeffer, Alexander Starke, Ard M. Nijhof, Sebastian Ulbert

**Affiliations:** 1 Department of Immunology, Fraunhofer Institute for Cell Therapy and Immunology, Leipzig, Germany; 2 Institute of Bacterial Infections and Zoonoses (IBIZ), Friedrich-Loeffler-Institut, Federal Research Institute for Animal Health, Jena, Germany; 3 Institute of Animal Hygiene and Veterinary Public Health, University of Leipzig, Leipzig, Germany; 4 Clinic for Ruminants and Swine, Faculty of Veterinary Medicine, University of Leipzig, Leipzig, Germany; 5 Institute for Parasitology and Tropical Veterinary Medicine, Freie Universität Berlin, Berlin, Germany

**Keywords:** *Coxiella burnetii*, Ticks, Transmission

## Abstract

**Background:**

The bacterium *Coxiella burnetii* is the etiological agent of Q fever and is mainly transmitted *via* inhalation of infectious aerosols. DNA of *C. burnetii* is frequently detected in ticks, but the role of ticks as vectors in the epidemiology of this agent is still controversial. In this study, *Ixodes ricinus* and *Dermacentor marginatus* adults as well as *I. ricinus* nymphs were fed on blood spiked with *C. burnetii* in order to study the fate of the bacterium within putative tick vectors.

**Methods:**

Blood-feeding experiments were performed *in vitro* in silicone-membrane based feeding units. The uptake, fecal excretion and transstadial transmission of *C. burnetii* was examined by quantitative real-time PCR as well as cultivation of feces and crushed tick filtrates in L-929 mouse fibroblast cells and cell-free culture medium.

**Results:**

Ticks successfully fed in the feeding system with engorgement rates ranging from 29% (*D. marginatus*) to 64% (*I. ricinus* adults). *Coxiella burnetii* DNA was detected in the feces of both tick species during and after feeding on blood containing 10^5^ or 10^6^ genomic equivalents per ml blood (GE/ml), but not when fed on blood containing only 10^4^ GE/ml. Isolation and cultivation demonstrated the infectivity of *C. burnetii* in shed feces. In 25% of the *I. ricinus* nymphs feeding on inoculated blood, a transstadial transmission to the adult stage was detected. Females that molted from nymphs fed on inoculated blood excreted *C. burnetii* of up to 10^6^ genomic equivalents per mg of feces.

**Conclusions:**

These findings show that transstadial transmission of *C. burnetii* occurs in *I. ricinus* and confirm that *I. ricinus* is a potential vector for Q fever. Transmission from both tick species might occur by inhalation of feces containing high amounts of viable *C. burnetii* rather than *via* tick bites.
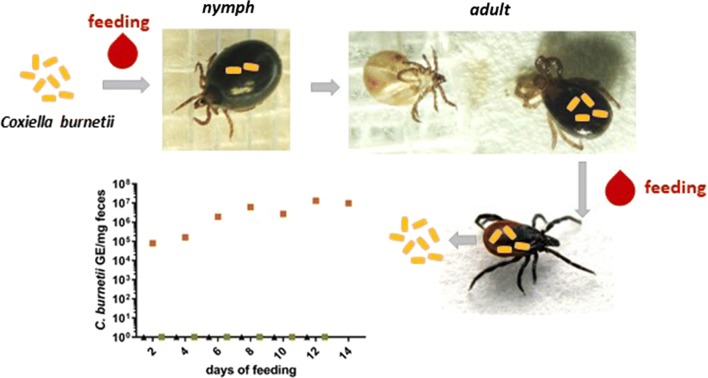

## Background

*Coxiella burnetii* is an obligate intracellular bacterium and distributed worldwide with the exception of New Zealand. It is known as the causal agent of Q fever and affects a wide range of hosts including humans [1, 2]. Infected ruminants, considered as the main reservoir, show no symptoms or fertility-related complications and can shed the bacteria in large amounts during parturition or defecation [3, 4]. Human Q fever infection is often presented as an acute flu-like disease with possible complications such as pneumonia or hepatitis [5, 6]. Moreover, a chronic form mainly affecting heart-valves as well as a fatigue syndrome can occur [7, 8]. The most relevant infection route of Q fever is the inhalation of contaminated dusts or aerosols. Persons with close contact to infected domestic ruminants, such as farmers or veterinarians, may be at higher risk for acquiring this disease [9, 10].

*Coxiella burnetii* was first isolated from an infected *Dermacentor andersoni* tick [11] and was then isolated from 40 different tick species, including *Ixodes ricinus*, the most common tick in central Europe [12–15]. The tick *D. marginatus* in particular is considered to be a competent vector. The spatial distribution of this tick matches the increased occurrence of Q fever cases in southwest Germany [16, 17]. In early studies, the presence of *C. burnetii* in tick organs was examined by staining methods [18, 19]. With the introduction of PCR methods, several studies investigating the prevalence of tick-borne pathogens in Europe showed *C. burnetii* DNA in over 10% of the examined arthropods [20, 21]. Contrary, in other studies, no *C. burnetii*-specific DNA was detected in ticks [22–24]. In addition, tick feces samples were found to contain *C. burnetii* or DNA of this agent [17, 25]. However, recent research questioned the role of ticks in Q fever transmission. During the large Q fever outbreak in the Netherlands 2007–2010, ticks obtained from environment, pets, wildlife and livestock were tested, but no infected tick could be found, even from infected herds [26]. Thus, the risk of acquiring Q fever by ticks is commonly assessed as being low. Furthermore, *Coxiella*-like bacteria were discovered as primarily non-pathogenic tick endosymbionts [27, 28]. Their close genetic relationship to *C. burnetii* could have led to misidentifications by PCR [29, 30]. These partially contradictory findings demonstrate the need for further research on possible transmission routes of *C. burnetii* between different life stages of ticks and from ticks to their hosts.

To study blood-feeding and vector competence of hematophagous arthropods under controlled laboratory conditions, several *in vitro* feeding systems have been established [31–35]. These methods allow detailed analyses of blood-borne pathogen transmission *via* insect or tick vectors. In this study, the uptake, survival and transstadial transmission of *C. burnetii* in ticks, as well as excretion of the bacteria *via* feces, was analyzed using a membrane-based *in vitro* feeding system.

## Methods

### Ticks

Adult female and male *I. ricinus* and *D. marginatus* as well as *I. ricinus* nymphs were obtained from a laboratory colony (Insect Services, Berlin, Germany) at the age of 14 to 37 weeks after molting. The colonies were free of *Borrelia* spp. and *Rickettsi*a spp. Until feeding, *I. ricinus* ticks were maintained at 4–8 °C and *D. marginatus* at room temperature at a relative humidity (RH) of > 90%. At least 24 h before the beginning of tick feeding experiments, *I. ricinus* were moved to room temperature.

### Membrane production

Artificial tick feeding was performed in silicone-membrane sealed glass tubes following the protocol by Kröber and Guerin [36]. Briefly, the silicone mass was mixed according to the recipe published by Krull et al. [37] and spread out on lens cleaning tissue (Whatman, Maidstone, UK) with the rounded side of a gel releaser (Bio-Rad, Hercules, USA). Membranes were left to polymerize for at least 16 h at > 95% RH. Membrane thickness was measured with the Inductive Dial Comparator 2000 (Mahr, Göttingen, Germany). Membranes with a thickness of 70–100 µm were used for the feeding of *I. ricinus* adults. *Ixodes ricinus* nymphs and adult *D. marginatus* were fed on membranes of 60–80 µm.

### Artificial feeding system

Sealed feeding units were made by gluing the silicone membrane with Elastosil E41 (Wacker Chemie, München, Germany) to borosilicate tubes of 50 mm length, 30 mm outer diameter, 28 mm inner diameter (Neubert Glas, Geschwenda, Germany). A square piece of mosquito netting (15 × 15 mm, Draht Driller, Freiburg, Germany) was loosely glued to the membrane [37] to stimulate the fixation of adult ticks. An extract of washed sheep wool (Alana, Karlsruhe, Germany) was prepared with dichloromethane (Carl Roth, Karlsruhe, Germany) as described by Böhme [38]. Ninety µl of the extract (10 mg/ml) was added to the membrane of each feeding unit, which were subsequently allowed to dry for 2 h at room temperature before ticks were placed in the feeding unit [38].

### Feeding of adult *I. ricinus*

Blood used for feeding experiments was taken from the jugular vein of cattle (Thüringer Landesamt für Verbraucherschutz, Bad Langensalza, Germany, registration no. 04-102/15) in heparinized blood vials (Sarstedt, Nümbrecht, Germany). Of this blood, 3.1 ml supplemented with glucose (2 g/l) were added to each well of a 6-well plate (Sarstedt). As a phagostimulant, 1 mmol/l ATP (Carl Roth) dissolved in physiological sodium chloride solution was added to the blood [39]. The plate was pre-warmed at 37 °C. Blood was changed every 10–14 h. The tick feeding units placed in the 6-well plates were incubated at 37 °C, a RH of > 70% and a day–night regime with a cycle of 15 h light and 9 h darkness. All environmental factors were continuously monitored by a data logger (MSR, Seuzach, Switzerland). As a further stimulus, the CO_2_ level was increased to 2.5% [40]. Each feeding unit contained 7–9 female and 5 male *I. ricinus*.

During the first 12 h of each feeding experiment, ticks were fed on blood (without bacteria) for attachment before starting inoculation. For the infection experiments 10^4^ (2 units), 10^5^ (2 units) or 10^6^ (5 units) genomic equivalents (GE)/ml of *Coxiella burnetii* Nine Mile phase II RSA 439 (obtained from the strain collection at Friedrich Löffler Institute Jena) were subsequently added to the wells of the feeding unit during every blood change, except for the mock infection control (2 units). In one of the experiments, attached females feeding on *Coxiella*-inoculated blood were removed from the membrane after 48 h of feeding and transferred to a fresh feeding unit in order to continue feeding on *Coxiella*-free blood (4 units). During each blood change, the membranes and ticks were inspected and feeding units were rinsed with warm 0.9% sodium chloride solution. Samples of tick feces of every feeding unit were removed daily with fine forceps and stored at 4 °C until further processing. Single ticks were removed during feeding at different days. Detached replete ticks were removed from the membrane, transferred to a 15 ml Falcon tube and individually stored at 21–23 °C, > 90% RH.

### Feeding of *D. marginatus*

Blood for feeding of *D. marginatus* adults was prepared the same way as for *I. ricinus* adults. The experiments were performed with addition of 10^6^ GE/ml *C. burnetii* in 4 feeding units or mock inoculation in 2 feeding units. Per feeding unit, 7 female and 5 male *D. marginatus* were used. Feeding chambers were incubated on a 38 °C heating plate with an ambient temperature of 28–30 °C. This provides a temperature gradient, resembling the animal skin and environmental temperatures. Blood change as well as storage of feces samples and engorged ticks was carried out as in *I. ricinus* adult experiments.

### Feeding of *I. ricinus* nymphs

The feeding of *I. ricinus* nymphs was performed in the same setting as for *D. marginatus*. CO_2_ was used at a concentration of 4% and the feeding units were covered with a moist piece of cotton wool to prevent desiccation of ticks. In each feeding unit, 50–60 nymphs were placed. One feeding unit remained as mock infected, whereas the other were constantly supplemented with 10^6^ GE/ml throughout feeding. Blood change was performed similar to the adult feeding and engorged nymphs were removed and transferred to 1.5 ml tubes and maintained at 21–23 °C and a RH of over 90%. Shortly after engorgement, 5 mock infected nymphs and 6 nymphs, which were exposed to *C. burnetii* were removed and tested by qPCR.

### Cultivation of *Coxiella burnetii*

All experiments with *C. burnetii* Nine Mile Phase II RSA 439 were carried out under biosafety laboratory 2 conditions. *Coxiella burnetii* was cultivated in L-929 mouse fibroblasts, maintained in Dulbecco’s Modified Eagle Medium (DMEM; Life Technologies, Carlsbad, USA) supplemented with 5% fetal calf serum (FCS; Life Technologies). Medium was changed weekly, and the cells were harvested after an average of two weeks when *Coxiella*-containing vacuoles were visible. Purification of bacteria from host cells was performed *via* needle purification using 22 G cannulas as smallest size according to [41], and genomic equivalent measurement was performed by quantitative real-time PCR as described below. *Coxiella burnetii* was stored in a 45 mM phosphate-buffered 0.25 M sucrose solution with 10% glycerol in aliquots at − 80 °C.

### DNA isolation and quantitative real-time polymerase chain reaction (PCR)

Female ticks from feeding experiments were stored in 70% ethanol at 4 °C. Prior to DNA-extraction, ticks were washed in PBS and dried. Every tick was weighed individually using a micro balance. In addition, feces per day and group were weighed in order to normalize the PCR results to the mass of feces. Lysis of ticks and feces was performed in a Tris-EDTA-lysozyme-buffer [42], containing 0.25 M Tris-HCl (pH 7.5), 10 mM EDTA and 0.4% (w/v) lysozyme, with micropestles (Eppendorf, Wesseling-Berzdorf, Germany) and in the case of ticks, an additional two rounds of centrifugation (14000×*g*, 5 min, 20 °C) in 1.5 ml tubes. After 4 h of lysis with 0.2 % (w/v) proteinase K at 56 °C, DNA extraction was performed according to the High Pure PCR Template Preparation Kit (Roche, Basel, Switzerland). In addition, DNA was extracted from 200 µl blood that was used to feed previously infected ticks with the same DNA extraction kit according the whole blood protocol. Quantitative real-time PCR was performed with a Lightcycler 480 II (Roche) targeting the *C. burnetii* single-copy gene *icd* [43]. For each reaction 2 µl of template DNA or standard was used [44]. The program included 45 cycles of 15 s at 95 °C and 30 s at 60 °C. A cut-off for positivity was set to a Cq-value of 35, which was equal to 10 copies per reaction. For confirmation of DNA extraction in *I. ricinus*, a quantitative real-time PCR targeting the *I. ricinus* housekeeping genes *actin* and *elongation factor 2* was conducted using the primers described in [45].

### Isolation of *Coxiella burnetii* from feces

Tick feces samples originating from one group fed on blood inoculated with 10^6^ GE/ml *C. burnetii* and from one mock infected group were weighed and used for cultivation in L-929 cells. Briefly, ninety-two mg feces from mock infected and 102 mg feces from infected ticks were dissolved in 10 ml DMEM containing 5% FCS, filtered through 0.45 µm syringe filters (TPP, Trasadingen, Switzerland). From the filtrate, 4.5 ml were transferred to a 25 cm^2^ flask (Greiner bio-one, Kremsmünster, Austria) of confluent murine L-929 cells. The volume was then extended to 6 ml with DMEM containing 5% FCS. Medium was changed weekly, and cells were visually monitored at 400× magnification. For PCR-based affirmation of the visible increase of vacuoles, every three days a square of approximately 1.5 cm^2^ of the cell layer was removed with PBS (pH 7.4) using a cell scraper. Cells were centrifuged (15000×*g* for 5 min at 4 °C) and the pellet resuspended in lysis buffer. DNA was extracted and *C. burnetii* DNA was quantified using the isocitrate dehydrogenase (icd) qPCR. For normalization the QuantiTect Probe RT-PCR Kit (Qiagen, Hilden, Germany) with the QuantiTect Primer for Ribosomal Protein L22 (*rpl22*) (Qiagen) targeting a mouse housekeeping gene was used, and the *icd* results were normalized to the average Cq values of *rpl22*.

### Isolation of *C. burnetii* from eclosed adults

Of the females molted from infected nymphs, two were bisected and qPCR was performed on one half whereas the other half was used for the cultivation of bacteria. For this purpose, halved ticks were suspended in 3 ml DMEM overnight before filtration through 0.45 µm syringe filters. One ml of this filtrate was used for inoculation of L-929 cells whereas the remaining filtrate was centrifuged at 15000×*g* for 5 min at 4 °C. Acidified citrate cysteine medium-2 (ACCM-2) was prepared as specified by the manufacturer (Sunrise Science, San Diego, USA). The pellet was resuspended in 2 ml ACCM-2 and placed into a 12-well plate. As a control, 2 ml ACCM-2 was inoculated with 10^5^ GE/ml *C. burnetii* RSA 439. The cultures were incubated at 37 °C with 5% CO_2_ and 2.5% O_2_ [46]. At day 1, 4 and 7, 100 µl were removed and used for qPCR quantification of *C. burnetii*. Inoculated L-929 cells were visually examined biweekly for signs of *Coxiella* containing vacuoles.

### Reinfection experiment

In order to examine transstadial transmission, females, which were fed as nymphs on *Coxiella*-containing blood, were fed with blood without *C. burnetii.* Therefore, two groups of 4 females and 3 males each were used. An additional uninfected group of the same composition was used as a negative control. Small feeding units (20 × 2.5 × 25 mm) were used in a 12-well-plate with 1 ml blood supplemented with 2 g/l glucose and of 1 mM ATP. During each blood change, 200 µl of blood was collected for qPCR analysis. Feces were removed every second day and also tested by qPCR and cultivation in L-929 cells.

### Statistical analysis

Data were analyzed using Excel to calculate the mean and standard deviation. SPSS Statistics V22.0 was used for performing unpaired two-tailed t-tests and Mann–Whitney U-tests, depending on data distribution. Pearson’s Chi-square test was performed for analysis of the engorgement rates. *P*-values < 0.05 were considered statistically significant.

## Results

### Behavior of *I. ricinus* and *D. marginatus* in the *in vitro* feeding assay

In five independent experiments a total number of 141 female *I. ricinus* were fed. Within the first 24 h of feeding, 58 ± 12% (mean ± standard deviation) of female *I. ricinus* ticks attached to the membrane. The attachment rate varied in different experiments between 84% and 100% at day 4. An engorgement rate of 49 ± 15% was achieved after 14.5 ± 3.1 days, as shown in Table 1. Adults feeding on inoculated blood took longer as for those taking an uninfected blood meal, but the difference was not statistically significant (Mann-Whitney U-test, *U* = 41.5, *Z* = − 1.453, *P* = 0.157).

**Table 1:** *In vitro* feeding of ticks

**Species**	Life stage	Engorgement rate (%)	Engorgement weight (mg)	Duration of feeding (days)	Time until oviposition	Molting rate (%)
*Ixodes ricinus*	Adults	49 ± 15	186.2 ± 61.8	14.5 ± 3.1	14.7 ± 4.9 days^a^	–
	Nymphs	~ 50	3.0 ± 1.0	7.9 ± 1.8	10 ± 5 weeks	92
*Dermacentor margiatus*	Adults	29	414.6 ± 136.7	14.8 ± 3.4	4 days^b^	–

^a^Days until oviposition of *I. ricinus* were calculated from 15 egg-laying ticks

^b^In the *D. marginatus* experiment, only 1 tick laid eggs after 4 days

*Notes*: Feeding parameters of *in vitro* experiments with *I. ricinus* adults and nymphs and *D. marginatus* adults (mean values ± standard deviation)

Visible feces production of ticks started at day 2 post-attachment. For DNA extraction, up to 40 mg of daily pooled feces per feeding group was used. During the rapid engorgement phase, which occurred after approximately 10 days of *in vitro* feeding, feces production decreased and the ticks engorged to an average weight of 186.2 ± 61.8 mg (average initial weight 2 mg).

The *in vitro* feeding assay was also established for *D. marginatus*. In two independent experiments fixation rates of 25% and 33% at day 4 and an engorgement rate of 29% (8 of 28 female *D. marginatus*) in both experiments were observed. This engorgement rate is lower than the one of *I. ricinus* adults, but the difference was not statistically significant (*χ*^2^ = 3.798, *df* = 1, *P* = 0.051). The engorgement weight was 414.6 ± 136.7 mg (average initial weight 8 mg) and engorged females had fed for an average of 14.8 ± 3.4 days. The engorgement weight of *D. marginatus* was significantly higher than the weight of engorged *I. ricinus* (*t*_(43)_ = −5 .356, *P* < 0.001). It was also noticed that females often detached and reattached during feeding.

Feeding was also performed with a total number of 200 *I. ricinus* nymphs. Approximately 50% of the nymphs engorged to an average weight of 3.0 ± 1.0 mg (average weight of unfed nymphs: 0.48 ± 0.31 mg) (Table 1). The remaining nymphs did not attach to the membrane or did not fully engorge. Nymphs fully engorged within 7.9 ± 1.8 days. After removal of 11 nymphs for PCR testing, within 10 ± 5 weeks 92% (82/89) of the engorged nymphs molted into adults, of which 59% were females.

### Infected *I. ricinus* can carry *C. burnetii* for at least seven weeks

To analyze the uptake of *C. burnetii* by ticks in the feeding system, 2–4 *I. ricinus* females exposed to 10^6^ GE/ml containing blood and one female tick exposed to negative blood were tested weekly for the presence of *Coxiella*-DNA, starting one week after beginning of feeding.

**Fig. 1:**
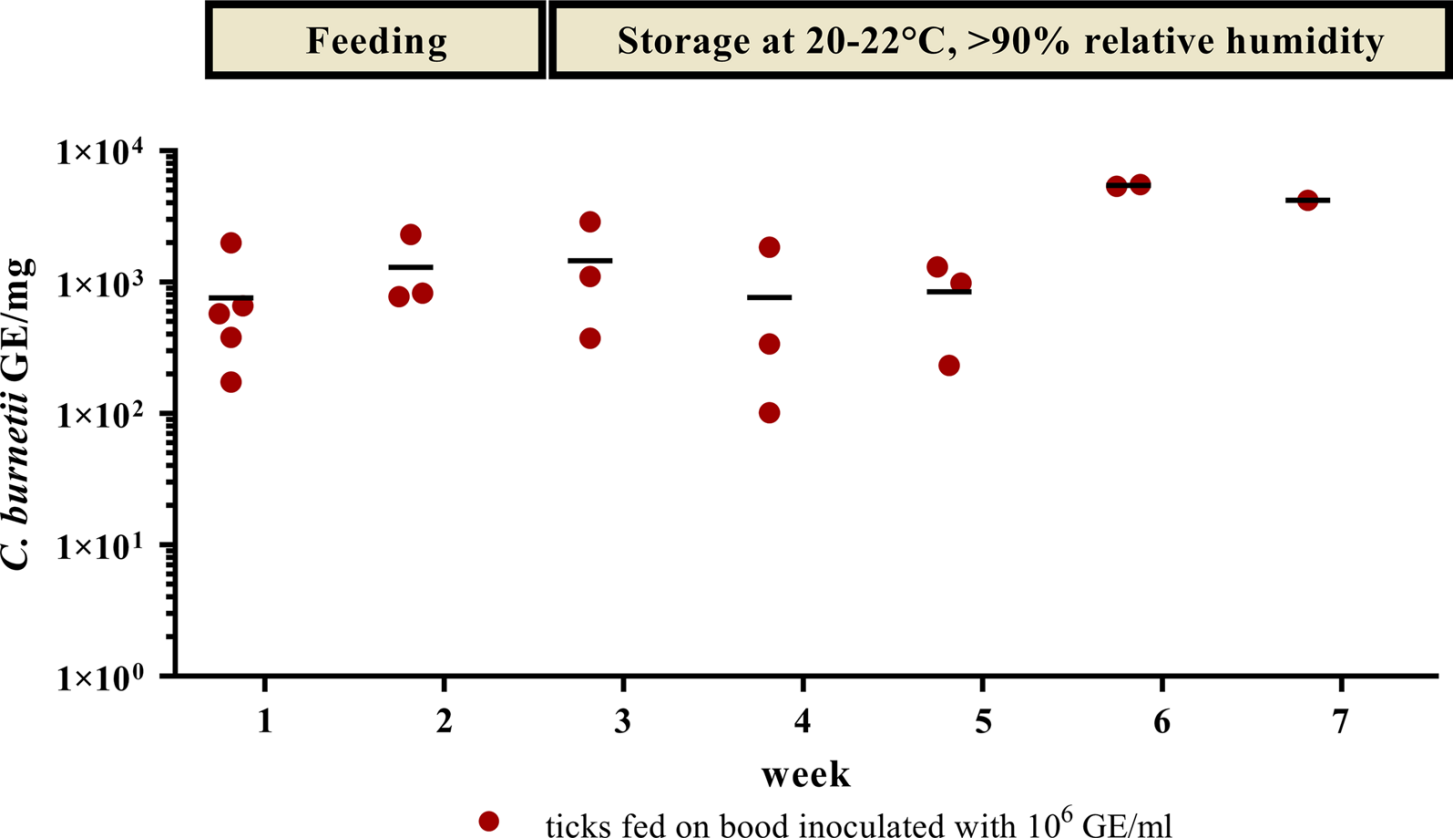
*Coxiella burnetii* DNA in ticks during and after feeding. Each red dot represents one tick, which was used for DNA extraction after feeding on blood inoculated with 10^6^ GE/ml of *C. burnetii* Nine Mile phase II. The means are indicated by a horizontal line. During the first two time points, the ticks were still feeding. Ticks were kept separated from each other after detaching from the membrane. Mock-infected ticks (one per time point, not shown) had no Cq-value. The data derive from two independent experiments and were normalized for the two *Ixodes ricinus* housekeeping genes *actin* and *elongation factor 2*

A slight but statistically not significant (*t*_(6)_ = − 0.969, *P* = 0.370) increase in *C. burnetii* DNA load was detected during feeding, i.e. in the first two weeks (Fig. 1). In all the female *I. ricinus* that fed on inoculated blood DNA of *C. burnetii* was found (ranging from 10^2^ to 10^3^ GE/mg) up to 5 weeks after feeding. In contrast, ticks feeding on negative blood remained *C. burnetii* negative (data not shown).

### *Coxiella burnetii* is excreted *via* feces by adult *I. ricinus* and *D. marginatus*

**Fig. 2:**
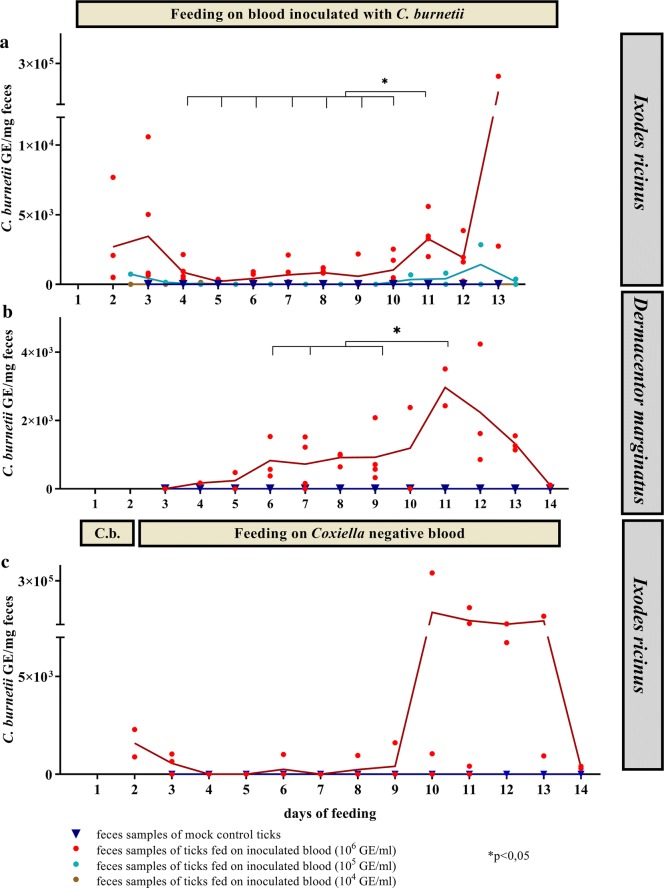
*Coxiella* DNA in feces. In (**a**) and (**b**) ticks were fed during the whole feeding experiment with blood inoculated with *C. burnetii* in different concentrations. The means of the replicates of each data set are connected with a line. The data for *I. ricinus* (**a**) represent three independent experiments with five different feeding units inoculated with 10^6^ GE/ml blood (*n* = 5), 10^4^ GE/ml (*n* = 2), 10^5^ GE/ml (*n* = 2) and negative controls (*n* = 2). For *D. marginatus* (**b**), the data represent two independent experiments with feeding units on blood inoculated with 10^6^ GE/ml (*n* = 4) and negative controls (*n* = 2). **c** Ticks were fed on 10^6^ GE/ml inoculated blood for 36 h (indicated by the horizontal bar labelled with ‘C.b.’) (*n* = 4). Attached ticks were subsequently removed and placed in a *Coxiella*-free feeding unit. Ticks constantly feeding on *Coxiella*-negative blood served as negative controls (*n* = 2). Statistical significance of DNA loads in comparison to day 11 is indicated by an asterisk

*Coxiella burnetii* DNA could readily be detected in feces of groups feeding on blood inoculated with 10^6^ GE/ml, but a decrease of the DNA content was observed after 3 days. However, after 9 days of feeding, the amount of *C. burnetii* DNA increased again. The DNA load at day 11 differed significantly from the DNA loads at the days 4–10 (e.g. *t*_(8)_ = − 4.631, *P* = 0.009 at day 5; *t*_(8)_ = − 2.768, *P* = 0.024 at day 10). Feces of ticks fed on 10^5^ GE/ml *Coxiella*-containing blood displayed a similar time-dependent excretion of bacteria (Fig. 2a), whereas a dose of 10^4^ GE/ml was not sufficient to obtain *Coxiella* DNA-containing feces. In *D. marginatus*, feces excretion started between day 3 and 6. In comparison to *I. ricinus*, larger amounts of feces were deposited, and also male *D. marginatus* attached and defecated for longer time periods. Similar to *I. ricinus*, in the feces samples examined by PCR the bacterial DNA load was highest between day 11 and 12 with up to 2 × 10^3^ GE per milligram feces (Fig. 2b) and was significantly different to the days 6 (*t*_(3)_ = 3.508, *P* = 0.039), 7 (*t*_(4)_ = − 3.413, *P* = 0.027) and 9 (*t*_(4)_ = − 3.024, *P* = 0.039).

To test whether shorter feeding periods would be sufficient for the excretion of *C. burnetii via* feces, the experimental setup was modified: *I. ricinus* were allowed to feed on *C. burnetii*-spiked blood for 36 h before being transferred to another unit where they continued feeding on bacteria-free blood. Here, a similar increase of *C. burnetii* DNA after approximately 11 days was observed in the feces (Fig. 2c), although the differences were not statistically significant.

**Fig. 3:**
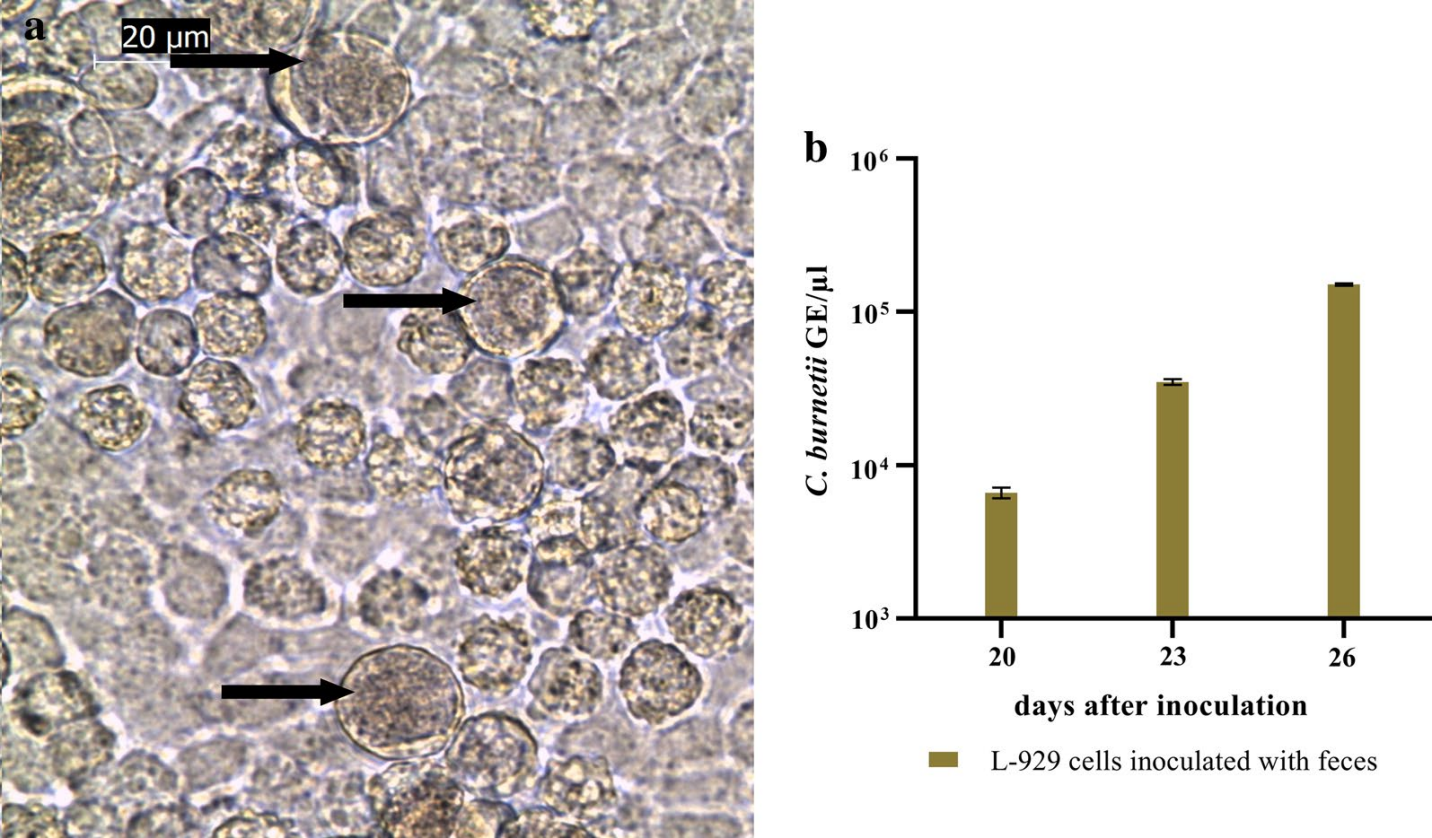
Growth of *Coxiella burnetii* from tick feces in L-929 cells. **a** *Coxiella*-containing vacuoles (indicated by arrows) 19 days after inoculation of L-929 cells with feces of ticks, fed for 36 h on *Coxiella*-containing blood. The picture was taken at 400× magnification. **b** Cells were removed from the flask at three different time points and genomic equivalents were determined by quantitative PCR. Samples were measured in duplicate, the mean values and standard deviation are indicated. The data were normalized for the mouse housekeeping gene *rpl22*

Next, we investigated whether the *C. burnetii* DNA in the tick feces derived from viable bacteria. Feces samples taken at day 10 from ticks fed on blood containing 10^6^ GE/ml (transferred to non-infected blood after 36 h, see above) were inoculated to L-929 cells. Samples from *Coxiella* negative blood-feeding were used as controls. In the cells incubated with feces from *C. burnetii* exposed ticks, first microscopically visible vacuoles appeared at day 18 after inoculation (Fig. 3a) and an increase in vacuoles was visible within the following ten days. At day 20, 23 and 26 after inoculation cells were harvested and analyzed by real-time PCR. An increase of *Coxiella* DNA in relation to the DNA of mouse fibroblasts (L-929) could be detected (Fig. 3b), whereas the flask inoculated with feces of negative controls remained free of *Coxiella* DNA. These data show that *C. burnetii* excreted in the feces from ticks feeding on infected blood are viable.

### *Coxiella burnetii* is transstadially transmitted from *I. ricinus* nymphs to adults

**Fig. 4:**
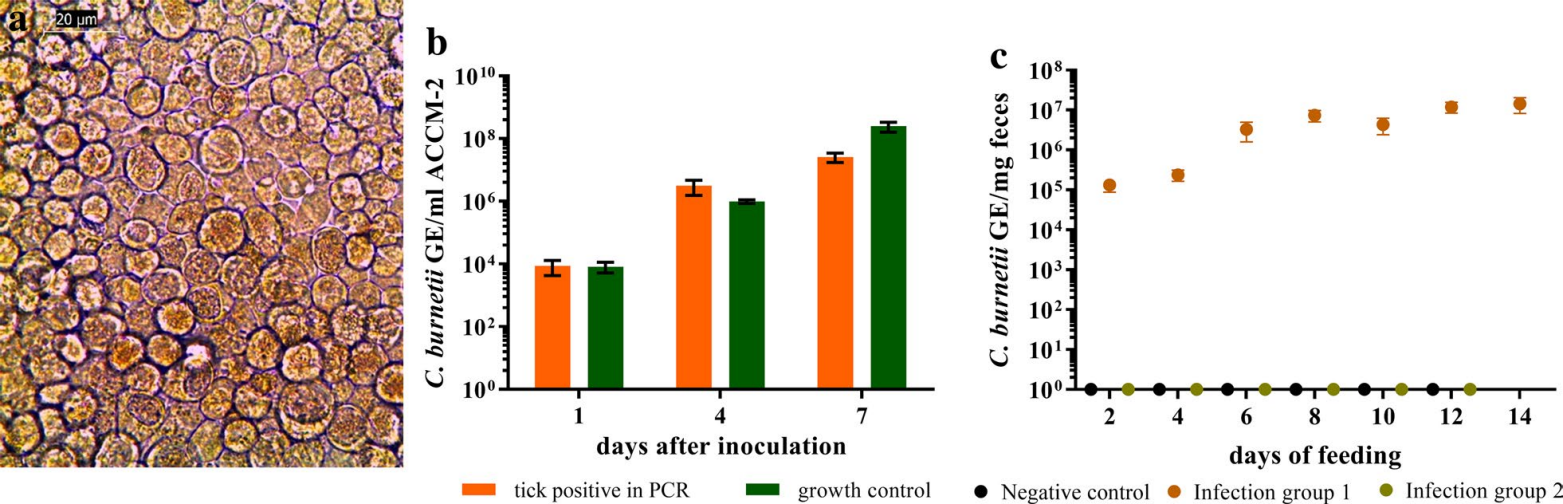
Transstadial transmission of *C. burnetii* from nymphs to adult *I. ricinus*. Nymphs were fed on blood containing 10^6^ GE/ml and were allowed to transform into adults. **a** Of the PCR *C. burnetii-*positive adult ticks, one half of a bisected tick was used for cultivation with L-929 cells. Vacuoles typical for *Coxiella* infections appeared 24 days after inoculation. **b** Material from the same tick was used for cultivation in cell-free ACCM-2 medium, and *C. burnetii* DNA was detected by PCR. Negative controls tested in parallel showed no Cq-value in the PCR (not shown). **c** Eight females molted from previously infected nymphs were divided into two infection groups, each consisting of 4 females and 3 *Coxiella*-negative males, and fed again on sterile blood. Feces were removed every two days and tested by quantitative PCR. Infection group 1 excreted *C. burnetii* DNA with their feces, whereas in feces of infection group 2 and in the negative control group no *C. burnetii* DNA was detected (here shown as 10^0^). Samples in **b** and **c** were analyzed in three independent measurements in duplicates, the mean values and standard deviations are indicated

In order to examine transstadial transmission of *C. burnetii*, *I. ricinus* nymphs were infected with 10^6^ GE/ml using the *in vitro* feeding system. *Via* qPCR, uptake of *Coxiella* DNA was confirmed by testing 6 previously infected nymphs, whereas 5 mock infected nymphs remained negative. Two weeks after molting, 40 adults, which were fed on *C. burnetii* inoculated blood as nymphs, and 26 adults fed on negative blood were tested by real-time PCR. Of the nymphs which were fed on inoculated blood 25% were positive for *C. burnetii* DNA. One female tick was bisected and one half was analyzed for *C. burnetii* DNA by PCR. The Cq-value was 22.69. After inoculation of axenic medium and L-929 cells with a filtrate of the other half of the tick, growth of *C. burnetii* in both settings was observed (Fig. 4a, b).

### *Coxiella burnetii* can be excreted *via* feces by adult ticks infected as nymphs

Eight female ticks which were fed with *C. burnetii* as nymphs were divided into two groups and fed again on sterile blood. In this reinfection experiment, all negative control ticks and 3 out of 4 ticks of each infection group were attached. In one of the infection groups, *C. burnetii* DNA was detected in feces at every time point, revealing an increasing DNA concentration up to 6 × 10^6^ GE/mg feces (Fig. 4c). The bacteria contained in the feces were infective as verified by culture in L-929 cells and PCR (Cq = 25.39) at day 21 post-inoculation. These results suggest that *C. burnetii* is transmitted transstadially from *I. ricinus* nymphs to adults and that infected adults excrete the bacteria with their feces during feeding on a non-infected host. Samples of blood on which these ticks had fed were tested at 7 different time points during the first 7 days of feeding, but *Coxiella* DNA was not detected.

## Discussion

The significance of ticks as potential vectors for *C. burnetii* is still under debate. Several laboratory-based studies have revealed that many tick species get infected upon feeding on *C. burnetii* positive animals and that they carry the bacterium into their next developmental life stage. In addition, transmission of *C. burnetii* to non-infected laboratory animals by ticks has been demonstrated [47]. Besides these principle findings, many questions remain unresolved, including the most significant mechanism of infection (e.g. *via* feces or saliva) or the effectiveness of transstadial transmission. It is important to investigate these aspects of *C. burnetii* in ticks, in order to fully understand the role these arthropods play in the epidemiology of Q fever.

In this study, we have investigated uptake of *C. burnetii* by *I. ricinus* and *D. marginatus via* blood-feeding. By using an artificial membrane feeding system it could be demonstrated that *I. ricinus* becomes infected by feeding on bacteria-containing blood and that the bacteria are detectable within the ticks for at least seven weeks. The feces, which are constantly being deposited during blood-feeding, contain viable *C. burnetii*. This excretion of *C. burnetii* was shown to be time dependent, as a peak of bacteria in feces was detectable at approximately 11 days upon start of feeding. The amount of *C. burnetii* found in the ticks and in their feces also correlated with the concentration of the bacteria in the blood, and was highest when feeding with 10^6^ GE/ml blood, the highest concentration used in this study. Titers of 10^6^ are commonly found in blood of humans with an acute Q fever infection [48]. Lower titers, 10^5^ GE/ml blood, created a similar bacteria excretion profile, whereas in the 10^4^ GE/ml-group *C. burnetii* DNA could not be detected, indicating the need of a highly bacteremic host for excretion *via* feces.

The appearance of *C burnetii* in tick feces 9 days after start of feeding on infected blood suggests a replication of *C. burnetii* in the cells of the midgut, confirming earlier studies [19, 25]. At the same time, we cannot exclude that this increase was in part or completely caused by the secretion of more concentrated feces during this period.

Tick feces are known as a potential source for infection *via* inhalation of infected dusts [49]. Our data suggest that, at least for *I. ricinus* and *D. marginatus*, tick feces are not always infectious but that the amount of active *C. burnetii* in feces strongly increases towards the end of the feeding period, provided that the tick was not already infected with *C. burnetii* before the blood meal (see below).

The use of an *in vitro* feeding system allows for well-controlled experimental conditions, especially for analyzing the effects of bacterial concentration and duration of feeding. In addition, no infection experiments with mammalian hosts are required. By using guinea pigs as hosts, Siroky et al. [50] demonstrated the principle vector competence of *Hyalomma aegyptium* for *C. burnetii*. However, the exact mechanisms of transmission could not be analyzed, which is possible with *in vitro* systems such as the one used in our study.

Our results demonstrate that adult ticks which became infected with *C. burnetii* as nymphs, contain viable bacteria and excrete them *via* feces during feeding on non-infected blood. This excretion of bacteria followed a different time course and contained higher concentrations as compared to non-infected ticks feeding on infected blood. DNA of *C. burnetii* was present in high concentrations from the start of feeding and did constantly increase during the blood meal. At the same time no bacterial DNA could be detected in the blood used for feeding of these adults. Although we cannot exclude that minimal amounts of *Coxiella* were secreted into the blood, given the detection limit of 10 GE per reaction [41], this finding might indicate that the primary route of transmission of *C. burnetii* by ticks is *via* feces. In the field, this transmission could occur in several ways. Dried feces can form infectious dusts which are inhaled by one or more potential hosts. Additionally, the feces can contaminate the bite wound caused by the tick, similar to transmission of the parasite *Trypanosoma cruzi* by reduviid bugs [51]. Likewise, *Rickettsia prowazekii*, the etiological agent of louse-borne epidemic typhus is only excreted through feces and not *via* saliva. The infection consequently takes place when the itching sting is scratched and the pathogen rubbed into the skin [52]. Also, the amount of feces might have influence on transmission efficiency. *Dermacentor marginatus* deposits larger amounts than *I. ricinus*, as observed by us (data not shown) and others [38], thereby secreting more bacteria.

From the *I. ricinus* nymphs which were fed on *C. burnetii*-infected blood, only about 25% were positive for the bacteria after molting into adults. This is in accordance to Siroky et al. [50], where 29% of adults which as larvae fed on *C. burnetii*-positive guinea pigs were infected. The result is also similar to data obtained with *in vitro* feeding systems and the tick-borne pathogens *Rickettsia monacensis* and *Anaplasma phagocytophilum* [35]. It indicates that transstadial transmission is not 100% efficient, but that roughly one out of four nymphs feeding on an infected host is able to transmit *C. burnetii* as an adult, under the selected experimental conditions. Accordingly, in our study, bacterial DNA in the feces was found only in one out of two experimental groups during the feeding of adults which were infected as nymphs. These results might contribute to the observation that ticks do not play a major role in transmitting Q fever in the field, although in principle they are competent vectors [47].

Our experiments were conducted with the Phase II Nine Mile strain of *C. burnetii*. This strain is non-pathogenic to humans due to a different LPS-structure when compared to the virulent Phase I strain [14]. The Phase II bacteria were chosen due to safety issues, as performing the *in vitro* assays requires extensive direct handling of infected ticks and their feces. However, we cannot exclude that the differences in surface structures could influence the behavior of the bacteria in the tick or in this *in vitro* feeding system. Therefore, key findings of our study should be repeated using virulent *C. burnetii* strains.

## Conclusions

This study provides evidence that the tick species *I. ricinus* and *D. marginatus* secrete viable *C. burnetii via* feces when feeding on infected blood. Moreover, approximately 25% of *I. ricinus* that became infected in the nymph stage still contained *C. burnetii* as adults and shed bacteria with their feces upon feeding on non-infected blood. As no *C. burnetii* was detected in the blood these adults fed on, our data support the idea that secretion *via* feces is a possible mechanism by which ticks can distribute and transmit *C. burnetii*.

## Data Availability

All data generated or analysed during this study are included in this published article.

## Abbreviations

ACCM: acidified citrate cysteine medium
GE: genomic equivalents
icd: isocitrate dehydrogenase
PBS: phosphate-buffered saline
PCR: polymerase chain reaction
RH: relative humidity
rpl22: ribosomal protein L22
